# Ultrasound-assisted water oxidation: unveiling the role of piezoelectric metal-oxide sonocatalysts for cancer treatment

**DOI:** 10.1007/s10544-024-00720-3

**Published:** 2024-08-19

**Authors:** Marco Carofiglio, Nicolò Maria Percivalle, Simelys Hernandez, Marco Laurenti, Giancarlo Canavese, Joana C. Matos, M. Clara Gonçalves, Valentina Cauda

**Affiliations:** 1 Department of Applied Science and Technology, Politecnico Di Torino, C.So Duca Degli Abruzzi 24, 10129 Turin, Italy; 2 CESAM - Universidade de Aveiro, Campus Universitário de Santiago, Aveiro, Portugal; 3 CQE, Centro de Química Estrutural, Universidade de Lisboa, Av. Rovisco Pais, IST, 1000 Lisbon, Portugal; 4 Departamento de Engenharia Química, Instituto Superior Técnico, Universidade de Lisboa, Av. Rovisco Pais, 1000 Lisbon, Portugal

**Keywords:** ZnO, Nanoparticles, Sonocatalysis, Piezoelectricity, Water oxidation, Cavitation

## Abstract

Ultrasound radiation has been widely used in biomedical application for both diagnosis and therapy. Metal oxides nanoparticles (NPs), like ZnO or TiO_2_ NPs, have been widely demonstrated to act as excellent sonocatalysts and significantly enhance cavitation at their surface, making them optimal for sonodynamic cancer therapy. These NPs often possess semiconductive and piezoelectric properties that contribute to the complex phenomena occurring at the water-oxide interface during sonostimulation. Despite the great potential in applied sonocatalysis and water splitting, the complex mechanism that governs the phenomenon is still a research subject. This work investigates the role of piezoelectric ZnO micro- and nano-particles in ultrasound-assisted water oxidation. Three metal oxides presenting fundamental electronic and mechanical differences are evaluated in terms of ultrasound-triggered reactive oxygen species generation in aqueous media: electromechanically inert SiO_2_ NPs, semiconducting TiO_2_ NPs, piezoelectric and semiconducting ZnO micro- and nanoparticles with different surface areas and sizes. The presence of silver ions in the aqueous solution was further considered to impart a potential electron scavenging effects and better evaluate the oxygen generation performances of the different structures. Following sonoirradiation, the particles are optically and chemically analyzed to study the effect of sonostimulation at their surface. The production of gaseous molecular oxygen is measured, revealing the potential of piezoelectric particles to generate oxygen under hypoxic conditions typical of some cancer environments. Finally, the best candidates, i.e. ZnO nano and micro particles, were tested on osteosarcoma and glioblastoma cell lines to demonstrate their potential for cancer treatment.

**Supplementary Information:**

The online version contains supplementary material available at 10.1007/s10544-024-00720-3.

## Introduction

The propagation of ultrasound (US) in liquid media and their interaction with both soft and hard matter is currently a hot research topic because of the wide applicability of ultrasound in several industrial and medical fields(Vilkhu et al. 2008; Carovac et al. 2011; Luo et al. 2021). In recent years, the introduction of nanostructured and high-surface area materials and their coupling with US have boosted the relevance of sonocatalysis for several applications ranging from environmental sciences to biomedical ones(Lops et al. 2019). For example, piezoelectric polymeric nanoparticles (NPs) were successfully used to fight glioblastoma multiforme when coupled with US stimulation(Pucci et al. 2022; Montorsi et al.). Metal oxides nanoparticles (NPs) with semiconductive properties have also gained a lot of attention in the field of cancer treatment thanks to their photocatalytic potential (photodynamic therapy)(Yi et al. 2020; Sargazi et al. 2022). However, the most employed semiconductive photocatalytic materials, i.e. titanium dioxide (TiO_2_) and zinc oxide, present some limitations under light stimulation, as a UV band gap(Dharma et al. 2022). This feature actually limits the efficacy of photodynamic therapy in deep-seated tumors, as UV-light cannot penetrate, necessitating further optimizations(Yu et al. 2009; Wang et al. 2009; Massaro et al. 2022). Therefore, the interest in the exploitation of US as an alternative and more penetrative way to activate nanostructured materials is growing fast(Cafarelli et al. 2021; Marino et al. 2024). The aim of this work is to explore the influence that different nanostructured oxides may have on the phenomena occurring in aqueous solutions during sonostimulation with a particular focus on the possibility to use these materials as catalysts for ROS and oxygen production in biological environments for cancer treatment. Indeed, the literature has already proved that sonodynamic therapy can be efficiently used for cancer treatment(Yan et al. 2020; Hu et al. 2022). In particular, sonodynamic therapy has been demonstrated somehow effective both with TiO_2_ and ZnO NPs against various cancer cells(Vighetto et al. 2021, 2024; Carofiglio et al. 2022; Racca et al. 2023). However, to our knowledge, few works studied nanoparticles-assisted sonostimulation of water as a method to perform water oxidation to generate oxygen for water splitting (Ikeda et al. 1999; Hong et al. 2010; Xu et al. 2020b), but no work systematically compared different oxides NPs with different properties to discriminate the chemical moieties produced at the NPs surface during sonostimulation in aqueous media and investigate it in view of a biomedical application.

The reason for the lack of studies in this specific niche of sonocatalysis could lie in the complexity of the phenomena occurring in a US-stimulated fluid. In general, the US stimulation of an aqueous solution triggers a series of effects mainly attributable to the establishment of inertial cavitation(Canavese et al. 2018). Periodic acoustic waves can in fact cause the oscillation and consequent collapse of gas micro-bubbles. During this collapse, a large part of energy is released and micro-environments with particularly harsh conditions are created causing numerous physical and chemical phenomena. Some of these are:

- Sonoluminescence: the generation of photons after the bubble collapse(Margulis and Margulis 2004; Crum 2015; Vighetto et al. 2022). These photons could potentially excite the metal-oxide semiconductor particles present in the solution, leading to a secondary photocatalytic effect. These photocatalytic effects could lead to a secondary photodynamic therapy in which the light source is localized in the tumor, avoiding light penetration limits of conventional photodynamic therapy.
- Water sonolysis: in the case of simple water, US stimulation can cause the generation of reactive oxygen species (ROS) upon water sonolysis(Canavese et al. 2018; Vighetto et al. 2019; Ancona et al. 2020).
- Sonochemical reactions or sonocatalysis: as definition, it is the acceleration of some chemical reaction by means of ultrasounds. This happens because cavitation may lead to very high local pressures and temperatures(Flint and Suslick 1991) which can accelerate otherwise very slow reactions.

Furthermore, in the systems considered in this study, the aqueous solution is loaded with particles which can not only influence the cavitation threshold of the liquid, but also actively contribute to the occurring phenomena(Ancona et al. 2020; Vighetto et al. 2022; Troia et al. 2023). In fact, as already mentioned, semiconductor particles can be excited by the light generated by the cavitating gas bubbles. Our hypothesis is that piezoelectric particles, such as ZnO nano and microparticles, can manifest an electromechanical effect when subjected to a pressure stimulus and contribute, with the generated charges on their surface, to the complex processes that occur in the US stimulated aqueous solution, finally leading to water oxidation.

In the present work, we aim to establish the phenomena that contribute to the US assisted water oxidation of metal-oxide containing solution and to determine whether the piezoelectricity and surface area of ZnO coupled with an ultrasonic stimulation can be exploited for this purpose. More in detail, the production of reactive oxygen species following the US exposure of an aqueous solution containing three metal oxides is measured. To clarify as much as possible the contribution to acoustic cavitation from the semiconductor and piezoelectric properties of the metal oxide particles, three different materials are chosen, according to their different physical properties: (i) electrical insulating silica NPs (SiO_2_ NPs) as inert system; (ii) semiconductive titania NPs (TiO_2_ NPs), as commonly used material in the field of water splitting catalysis; (iii) semiconductive and piezoelectric ZnO NPs, as potential catalysts for ultrasound assisted water oxidation. Furthermore, two alternative ZnO microparticles morphologies (desert-roses and micro-wires) were also considered and compared to the ZnO NPs, to understand whether the morphology and surface area of the particle has a role in the reactive oxygen species production. All the particles were also analyzed by dissolving silver nitrate salts (AgNO_3_) in the aqueous solution: silver nitrate is indeed typically employed in photocatalytic experiments in combination with the semiconductor catalyst, as it stabilizes the possible electrons promoted to the conduction band from the valence band and inhibits their recombination with the holes acting as electrons scavenger. Thus, AgNO_3_ energetically promotes water oxidation through the holes, as commonly performed in photocatalysis set-ups and maximizes the effects that can be present in biological media where a large number of ions are present.

By evaluating the production of ROS by electron paramagnetic resonance (EPR) spectroscopy, and by characterizing the particles before and after sonostimulation, we gain the understanding of a possible mechanism to unravel the water oxidation reaction from the US-assisted piezocatalytic activity of ZnO particles.

 The production of oxygen resulting from NP-assisted ultrasound (US) stimulation of aqueous solutions was measured, adopting osteosarcoma and glioblastoma as models of deep-seated tumors to test the efficacy of the treatment. The data showed a clear correspondence between results obtained from EPR spectroscopy and gas chromatography.

This proof of concept highlights the potential of combining semiconductor and piezoelectric ZnO particles with US stimulation against hypoxic cancers obtaining an oxygen and ROS producing weapon.

## Materials and methods

### Oxide synthesis

ZnO NPs with a nominal diameter of 20 nm were purchased from Io⋅li⋅tec nanomaterials. Silica NPs (SiO_2_ NPs) of approximately 20 nm of diameter were synthetized following the Stöber method(Stöber et al. 1968; Bogush et al. 1988) in a similar way to what done in another work(Vighetto et al. 2022). In this particular case, 0.558 mL of tetraethyl orthosilicate (TEOS, ≥ 99% Sigma-Aldrich) was dispersed in 25 mL of ethanol (99%, Merk) in a plastic 50 mL centrifuge tube. The solution was vigorously stirred for 15 min. 1.018 mL of ammonium hydroxide solution (NH_4_OH, ACS reagent, 28–30%, Sigma-Aldrich) was rapidly added to the solution to start the nucleation process. The solution was left in moderate stirring for 25 h, time after which the solution become opalescent. The dispersion was then collected and centrifuged (12000 g for 20 min). The particles were redispersed in ethanol. This washing step was repeated twice to remove all the non-reacted reagents.

Amorphous titania NPs (TiO_2_ NPs) were obtained following a procedure already reported in the literature(Matos et al. 2020; Vighetto et al. 2021). Sodium silicate solution in water (280 μL, Na_2_O⋅SiO_2_, 27% wt. % SiO_2_, Sigma-Aldrich) was diluted in 25 mL of ethanol and stirred for 15 min. To this solution, ammonium hydroxide in ethanol (25% w/w) was added and mixed for 15 min. Afterwards, the solution was placed into an ultrasound bath (TELSONIC, Bronschhofen, Tec-15, Economy-Cleaner) and 835 μL of titanium IV isopropoxide (TiPOT, Ti[OCH(CH_3_)_2_]_4_, 97%, Sigma-Aldrich) was added. The solution was sonicated for 30 min. The NPs dispersion was finally centrifuged for 15 min at 5600 g and resuspended in bidistilled water (obtained by a Direct Q3 system, Millipore) for four times.

ZnO microparticles with a desert rose like morphology (DR-ZnO) were synthetized with a sol–gel hydrothermal method, well established in the literature(Lops et al. 2019; Laurenti et al. 2020; Carofiglio et al. 2021). Briefly, zinc nitrate hexahydrate (0.49 M, Zn(NO_3_)_2_⋅6H_2_O, Sigma-Aldrich) and potassium hydroxide (0.99 M, KOH, Sigma-Aldrich) were separately dissolved in 100 mL of bidistilled water each. Then, the zinc nitrate solution was slowly dropped into the KOH solution. The overall mixture was stirred vigorously during all the process. After a white gel has formed, the solution was placed in an oven and treated at 70 °C for 4 h. DR-ZnO microparticles were then collected by filtration through a 2 μm particle retention filter and washed with bidistilled water. They were finally left drying overnight.

ZnO microwires (MW-ZnO) were synthetized with a procedure similar to the one used for the DR-ZnO microparticles, with a different amount of KOH (100 mL at 5.96 M) aimed at changing the molar ratio between the precursors and leading to a different morphology, as reported in a work from some of us(Cauda et al. 2015).

### Metal oxides characterization

All metal oxides micro and nanoparticles were characterized before and after US-stimulation in terms of crystallinity, morphology, composition, and optical properties. The morphology of the resulting particles was analyzed by means of field emission scanning electron microscopy (FESEM). In detail, metal oxides NPs were deposited onto a silicon substrate and attached to an aluminum sample holder. The samples were analyzed by means of a SUPRA 40 microscope (Zeiss) equipped with a detector for energy-dispersive X-Ray spectroscopy (EDS, x-act 10 mm^2^ Silicon Drift Detector, Oxford Instruments). The same specimens were also investigated in terms of elemental composition with EDS analysis. For EDS analysis only, silica NPs before and after US stimulation were deposited onto an aluminum foil, to exclude the presence of silicon due to the substrate in the measurement.

The considered metal oxides were also characterized in terms of crystalline structure by means of X-ray diffraction (XRD) analyses. Samples were prepared on a silicon substrate, as for FESEM analysis, and their diffraction patterns were obtained through a Panalytical X’Pert diffractometer in θ-2θ Bragg–Brentano mode (Cu-Kα radiation source, λ = 1.54 Å, 40 kV, 30 mA).

The oxides were also optically characterized. To do so, the fluorescence excitation and emission spectra of the oxides were acquired on aqueous suspensions of particles (3 mL at a concentration of 1 mg/mL) by means of a Perkin Elmer LS55 fluorescence spectrometer. Moreover, the optical absorption of the oxides was measured on a similarly prepared aqueous solution for the oxides both before and after sono-irradiation. The measurements were performed in transmission mode in the 200–1000 nm region of the light spectrum through a double-beam Varian Cary 5000 UV–vis-NIR spectrophotometer.

### Reactive oxygen species generation analysis

To analyze whether ROS are generated during the sonostimulation, electron paramagnetic resonance spectroscopy (EPR) coupled with the spin-trapping technique, was exploited. To perform these measurements, 1.82 mg of powder was dispersed in 2 mL of bidistilled water obtained by a Direct Q3 system (Millipore). All the measurements were also repeated by dispersing the metal oxide powders into a 8.5 mg/mL of silver nitrate (AgNO_3_, Sigma-Aldrich) water solution of equivalent volume, acting as electron scavenger. To the solution, 5,5-dimethyl-1-pyrroline-N-Oxide (DMPO, Sigma-Aldrich) was added as chemical trap, to obtain a final concentration of 10 mM. The 2 mL solution was withdrawn and introduced into a 24-well plate (Thermo Fisher). The system was stimulated by a piezoelectric ultrasound transducer (LipoZero G39, Globus), continuously at 1 MHz for 10 min. A small portion of the sonostimulated volume was withdrawn and analyzed by means of an EMXNano X-Band spectrometer (Bruker). The obtained signals were post-processed with the Bruker Xenon software (Bruker).

On similarly prepared samples, a fluorescent probe aimed at the detection of singlet oxygen was also considered. Singlet Oxygen Sensor Green (SOSG, Sigma Aldrich) was added at 1 µM concentration to the samples before their sonication. 2 mL of particles containing solution both with and without AgNO_3_ were stimulated similarly to what done for the EPR measurements. The fluorescence of 100 µL of solution at 528 nm was measured after exciting the samples at 485 nm with a Synergy HTX microplate reader (Agilent). Three technical replicas were measured and their fluorescence intensity mediated. The net intensity obtained was divided by the intensity of their respective non-stimulated medium (water or AgNO_3_ solution) and plotted as fold induction of that value.

### Sonocatalysis setup and experiments

The metal oxides were evaluated as potential sonocatalysts for the ultrasound-assisted water oxidation and consequent O_2_ production in hypoxic environments by exploiting a custom bubbling reactor coupled with a micro-Gas Chromatograph (μGC, Varian 490-GC equipped with a 10 m Molsieve 5A column and a micro-TCD, injection time of 40 ms and column temperature of 80 °C) as reported in previous works(Armandi et al. 2013; Thalluri et al. 2013, 2014; Hernández et al. 2014, 2018). More in detail, the reactor is composed of a 185 mL total volume borosilicate glass reactor (internal diameter 40 mm) equipped with an external jacket (4 mm of thickness) used to maintain a constant temperature through a 15 °C cooling water flow. On the top opening of the bubbling reactor, a Teflon® cap is used to seal the system. Two holes were used to inflate Argon (Ar, controlled by a Bronkhorst Mass Flow Controller) through of a stainless-steel tube (1/16’’ outside diameter) and to let the gas go toward the μGC respectively. A pressure transducer and a back-pressure regulator were exploited to maintain a constant relative pressure of 100 mbar in the reactor during the whole measurement by constant monitoring through a LabVIEW platform. A schematic of the setup is reported in Figure S1 (Supporting Information).

The measurements were conducted as follows. First, 100 mg of powder was dispersed in 110 mL of bidistilled water obtained by a Direct Q3 system (Millipore). All the measurements were also repeated by dispersing the metal oxide powders into a 935 mg of silver nitrate (AgNO_3_, Sigma-Aldrich) water solution of equivalent volume, acting as electron scavenger. The particles suspension was placed into the bubbling reactor and sealed with the Teflon cap. The cooling system was turned on and the reactor was placed into an ultrasound bath operating at 59 kHz (Branson 3800 CPXH, Branson Ultrasonics Corporation). Before the sonostimulation, Ar at 50 NmL/min was fed in the liquid dispersion up to complete air stripping. When the outlet air content, measured through the μGC, reached a plateau, the Ar flow rate was brought to 6 NmL/min and kept constant during the whole measurement, with also a constant pressure of 100 mbar. The oxygen content measured in these conditions was considered as baseline of the measurement and subtracted from the evaluation of the total oxygen generation.

The oxygen concentration of the gas inside the reactor chamber was evaluated over time from the moment in which the ultrasound stimulation was turned on. The total stimulation lasted 1 h for all the considered oxides. Gas analyses were performed every 70 s. The amount of oxygen measured by the μGC was exploited to calculate the oxygen flux Φ(O_2_) by means of the formula(Armandi et al. 2013):

1 $$\Phi \left({O}_{2}\right)=\frac{\left(\frac{{C}_{{O}_{2}}}{1-{C}_{{O}_{2}}}\right)P{Q}_{Ar}}{RT}$$

where ${C}_{{O}_{2}}$ is the measured oxygen concentration, P is the pressure inside the reactor, Q_Ar_ is the Argon flow rate, T is the temperature in the system (kept constant at 17 °C during the measurement) and R is the ideal gas constant.

The result was numerically integrated over time to obtain the total amount of oxygen produced during sonostimulation. All the measurements were compared to the amount of oxygen obtained during the sonostimulation of water alone (without the AgNO_3_ scavenger) by subtracting the amount obtained in the latter case with the actual measurement, to evaluate the produced oxygen excess.

### Live/Dead assay

The efficacy of combined ZnO particle and ultrasound (US) stimulation against cancer cells was evaluated using an osteosarcoma cell line (MG-63 (ATCC)) and a glioblastoma cell line (T98G (ATCC)) as a proof of concept via a Live/Dead assay. Cells were seeded at 40,000 cells/well in a 24-well TC-treated plate with 1 mL of cell culture medium (McCoy 5A medium (ATCC) supplemented with 10% Fetal Bovine Serum (FBS, ATCC) and 1% penicillin–streptomycin (P/S, Sigma-Aldrich) for MG-63 and DMEM (Sigma-Aldrich) with 10% of FBS, 1% of P/S and 2% of L-glutamine (Sigma-Aldrich) for T98G). After 24 h, three solutions containing 8 µg/mL of ZnO NPs, DR-ZnO, and MW-ZnO were prepared. The medium was replaced with 2 mL of particle-containing medium. After another 24 h, the medium was replaced with FBS-free medium, and cells were sonicated at 0.6 W/cm^2^ for 1 min. Subsequently, 200 µL of FBS-free medium containing Calcein AM (10 µM, Thermo-Fisher) and Propidium Iodide (1 µM, Thermo-Fisher) were added to each well for live/dead cell staining. After 30 min of incubation, cells were analyzed using a Nikon Ti-E inverted spinning disk confocal microscope with Spectra X Lumencor light sources, a 20 × objective, and an Andor Zyla sCMOS camera. Cell viability was assessed by counting live cells and dividing by the total number of cells in five images per condition.

## Results and discussion

### Metal oxide characterization

**Fig. 1:**
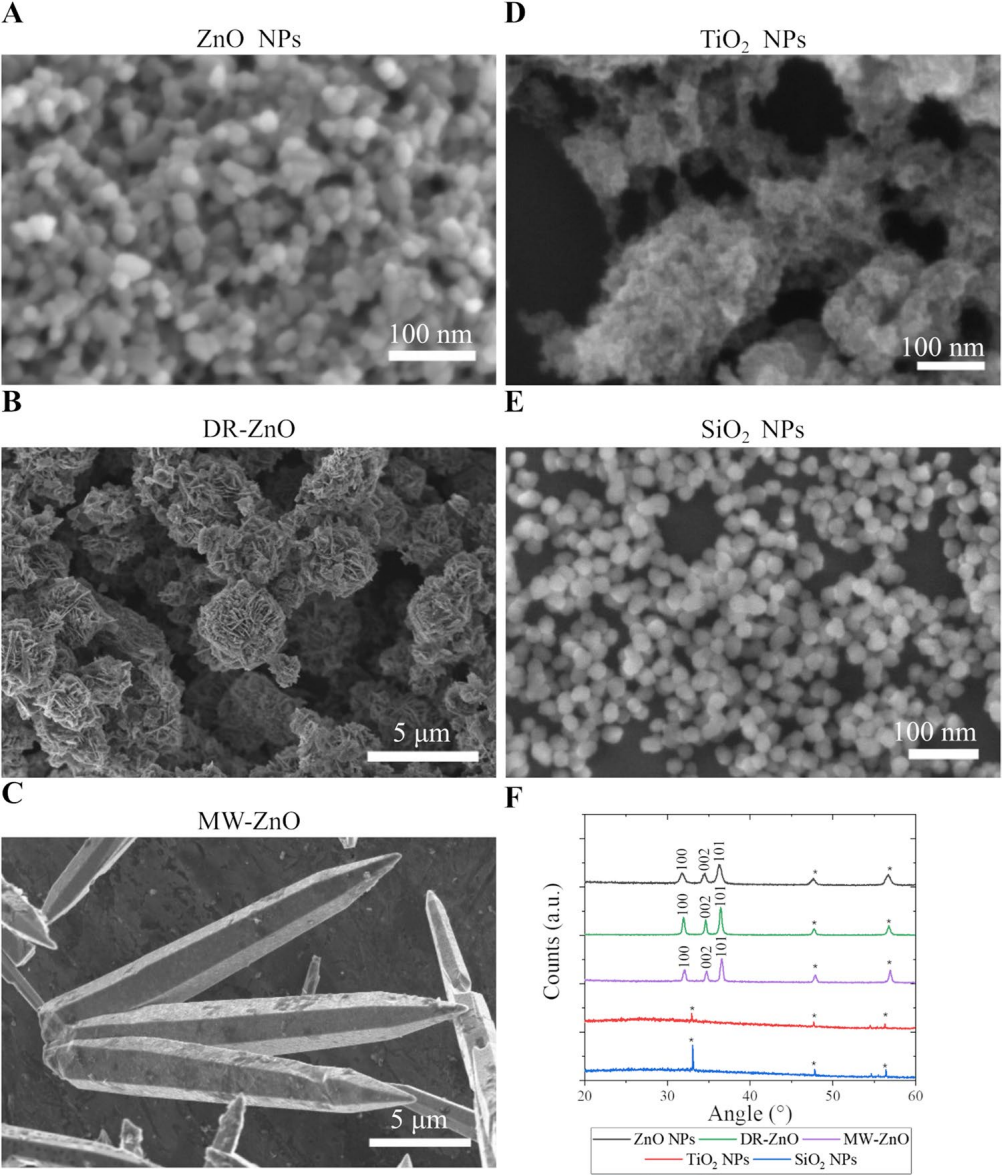
FESEM images of (**A**) ZnO NPs, (**B**) DR-ZnO microparticles, (**C**) MW-ZnO microparticles, (**D**) TiO_2_ NPs and (**E**) SiO_2_ NPs. (**F**) Analyzed oxides XRD patterns. * Refers to the peaks belonging to the silicon substrate onto which the substrates were deposited

Metal oxides NPs were characterized in terms of morphology by means of FESEM. As it is possible to observe from the images reported in Fig. 1, the oxides show different morphologies. More in detail, commercial ZnO NPs (Fig. 1A) are characterized by an overall spherical morphology with some irregularities. The nanocrystal diameters of approximately 20 nm confirm the manufacturer specifications. DR-ZnO microparticles can be considered as spherical, with a diameter between 3 and 4 µm composed by compenetrating lamellae exposing a high surface area (Fig. 1B). The morphological analysis of MW-ZnO microparticles reveals wires with length of several tens of micrometers and a transversal hexagonal geometry, with dimension of approximately 5–8 µm (Fig. 1C). In previous works from our groups, the three different morphologies of ZnO particles were characterized in terms of surface area, highlighting a of approximately 20, 5 and 1 m^2^/g for the DR-ZnO ZnO NPs and MW-ZnO particles respectively(Cauda et al. 2015; Lops et al. 2019). TiO_2_ NPs (Fig. 1D) are more irregular and characterized by a main dimension below 5 nm, similarly to what reported in another work in which the same synthesis technique was carried out(Matos et al. 2019). Finally, SiO_2_ NPs are found to be quite regular spherical NPs with a diameter of approximately 20 nm (Fig. 1E).

The particles elemental composition was determined by EDS analysis (Table 1). The results show 1:1 Zn:O stoichiometry, with an O excess that could be attributed to small surface contaminations or to the native layer of silicon oxide on top of the silicon substrate. In the case of TiO_2_ and SiO_2_ NPs, a stoichiometry of 3:1 was found (between O:M where M is the metal), indicating a larger amount of oxygen, which however could be attributed to the residuals coming from the synthetic technique.

**Table 1:** EDS analysis of the considered metal oxides expressed as atomic percentages

	Zn (At. %)	Ti (At. %)	Si (At. %)	O (At. %)
ZnO NPs	48.16 ± 0.26	-	-	51.84 ± 1.03
DR-ZnO	41.09 ± 1.47	-	-	58.91 ± 1.47
MW-ZnO	42.33 ± 5.73	-	-	57.67 ± 5.73
TiO_2_ NPs	-	23.18 ± 3.82	-	76.87 ± 3.82
SiO_2_ NPs	-	-	22.75 ± 1.83	77.25 ± 1.62

X-ray diffraction analyses allowed to investigate the crystallographic nature of the particles. From the XRD patterns reported in Fig. 1F it is possible to recognize the wurtzitic crystallographic structure of all the ZnO particles. Indeed, they all present peaks at the diffraction angles reported in JCPDS-ICDD (card n. 89–1397). There are differences in the width of those peaks among the different particles, which indicate a different crystallite size and can be attributed to the diverse synthesis method and dimension of the particles.

The TiO_2_ NPs present an amorphous crystalline structure since no peaks were observed in the XRD pattern. This result is in perfect agreement to what was found by Matos et al. for similarly obtained particles(Matos et al. 2020; Vighetto et al. 2021). A similar consideration can be done for SiO_2_ NPs, which predictably present an amorphous structure.

In this view, the analyzed particles can be separated into two distinct groups according to their crystalline structure, with the ZnO particles all showing a defined lattice structure and both titania and silica presenting an amorphous one.

**Fig. 2:**
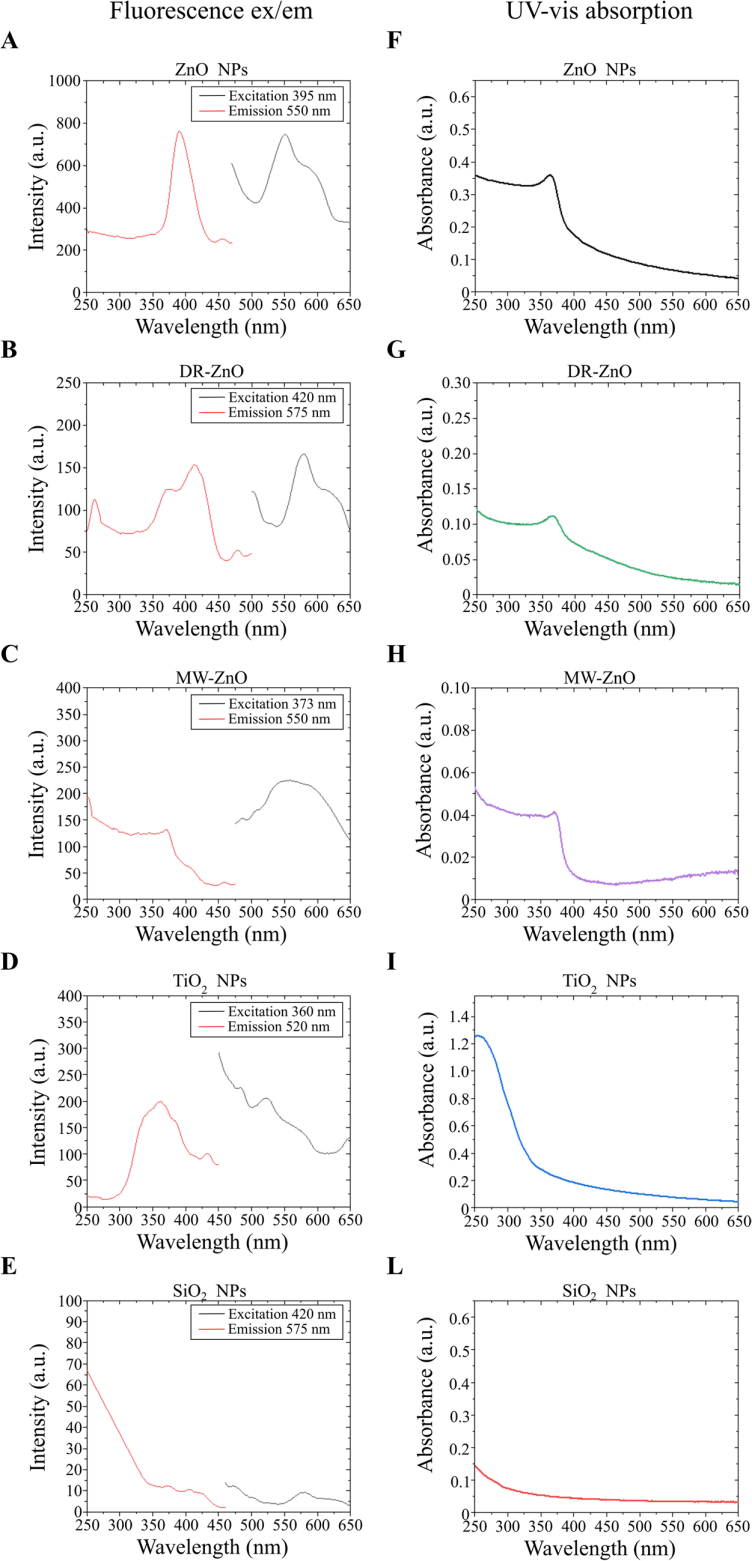
Fluorescence excitation (in red) and emission spectra (in black) acquired at specific emission and excitation wavelengths, respectively, for: (**A**) ZnO NPs, (**B**) DR-ZnO microparticles, (**C**) MW-ZnO microparticles, (**D**) TiO_2_ NPs and (**E**) SiO_2_ NPs. UV–vis absorption spectra acquired, respectively, for: (**F**) ZnO NPs, (**G**) DR-ZnO microparticles, (**H**) MW-ZnO microparticles, (**I**) TiO_2_ NPs and (**J**) SiO_2_ NPs

The studied metal-oxides NPs were optically analyzed to determine which are the differences among the various particles in terms of optical response and insulating/semiconductive behavior. This aspect is of foremost importance in the field of photocatalysts-assisted water oxidation, being the promotion of the metal-oxide electrons from the valence band to the conduction band the most important triggering phenomenon through which water oxidation occurs in photocatalysis(Yang et al. 2013; Xu et al. 2020a) and consequently in photodynamic. A complete understanding of the behavior of the particles under study is essential to determine the catalysis processes that occur during sonostimulation. Figure 2A-E reports the light excitation and emission spectra of the analyzed oxides. In particular, the red curve shows the intensity of the excitation spectrum when collecting the emission at the wavelength indicated in the legend (Emission λ nm), while the black curve represents the emission spectrum detected upon the excitation at the wavelength reported in the legend (Excitation λ nm). The ZnO particles, despite the differences in the excitation and emission peaks among the three samples, which could be attributed to the different crystallite sizes and particles dimensions, present a common behavior (as it can also be seen in figure S3 of SI). Indeed, they all show an excitation peak close to the 370–420 nm region of the light spectrum when collecting the emission signal at 550 nm. Furthermore, an emission peak in the 500–600 nm region is found when the particles are excited at each respective wavelength corresponding to the excitation maxima. More in detail, the ZnO NPs present an excitation peak at 395 nm and emission in the 510–560 nm region. DR-ZnO microparticles show the maximum excitation peak at 420 nm which results in an emission in the 550–600 nm region of the light spectrum. Finally, MW-ZnO are the particles presenting the lowest excitation wavelength peak among the analyzed ones (close to 373 nm), with a consequent emission in the 520–575 nm region. For what concerns TiO_2_ NPs, there is an emission peak at approximately 520 nm, which is maximum when the exciting light presents a wavelength of 360 nm. SiO_2_ NPs do not present any relevant peak and can be considered optically inert.

The metal-oxide micro and NPs were also analyzed in terms of optical absorption in the UV–vis range. Figure 2F-L highlights the optical similarities between the ZnO micro and NPs, with a relevant absorption in the UV region of the light spectrum and a rapid decay of the light absorption for wavelengths above 400 nm. However, the bandgap evaluation by the Tauc’s plot (Figure S2 in the Supporting Information) leads to the recognition of shifts in the optical absorption. Indeed, with a pattern similar to what found for the fluorescence spectra, the MW-ZnO particles present the highest bandgap (3.27 eV), followed by ZnO NPs (3.18 eV) and DR-ZnO particles (3.09 eV). All these particles present a lower band gap with respect to the one found for bulk ZnO (3.37 eV)(Özgür et al. 2005), probably because of the nanostructured morphology that characterizes all the considered particles. The TiO_2_ NPs present an even lower band gap (2.99 eV), which is similar to the band gap found for the rutile phase of bulk TiO_2_ (3.0 eV)(Chen and Mao 2007; Gonçalves et al. 2018), even if in this case the crystalline structure of the NPs is amorphous. Finally, SiO_2_ NPs presents a large band gap (6.04 eV), highlighting the insulating behavior of this material.

Despite small peak shifts that are of course attributed to the different nature of the considered semiconductor, here we present oxides having basically two different electronic behaviors. In one case, the SiO_2_ NPs, an electrically insulating system with optical inertness. In the second case, a group including semiconductor particles, i.e. all the ZnO particles and TiO_2_ NPs. Indeed, both ZnO and TiO_2_ present a strong excitation by UV light and an emission of visible light which has been already proven to be extremely useful when considering these systems for light-assisted catalysis(Morrison and Freund 1967; Lachheb et al. 2002; Malato et al. 2009; Ong et al. 2018). This emission could be potentially exploited for sonodynamic therapy, taking advantage of secondary phenomena triggered by US stimulation, like the sonoluminescence(Vighetto et al. 2022).

### Reactive oxygen species generation

The EPR spectroscopy was exploited to assess ROS generation during the ultrasound stimulation of the aqueous solution, using the DMPO spin trap. Also, the measurements were repeated in presence of a salt, AgNO_3_, which dissociates in Ag^+^ and NO_3_^−^. This salt was included to act as a scavenger of possible electrons promoted from the valence band to the conduction band of the semiconductors: in this way, electrons should be inhibited to recombine with the holes and this would energetically promote water oxidation through the holes.

**Fig. 3:**
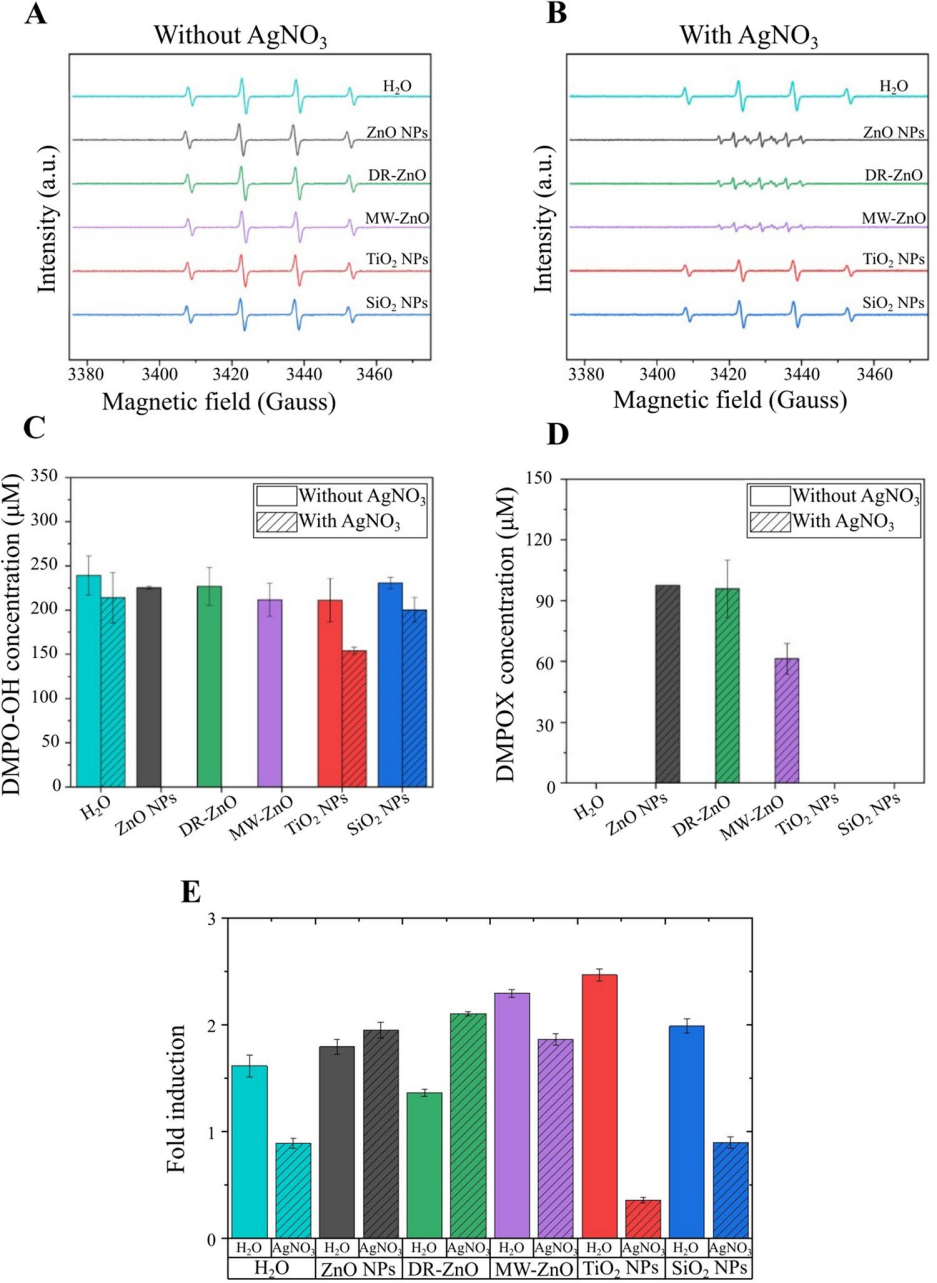
EPR spin adducts generated after ultrasound stimulation of the metal oxides in water dispersion without (**A**) and with (**B**) the scavenger in solution. Concentration of (**C**) DMPO-OH and (**D**) DMPOX species produced from water suspensions of metal oxides stimulated with ultrasound in presence or absence of electron scavenger. (**E**) Fluorescence intensity of the ultrasound stimulated oxide particles dispersion in presence of SOSG. The intensity is plotted as fold induction of not-stimulated H_2_O and AgNO_3_ solutions

Figure 3A-B shows the characteristic spectra derived from these measurements. In absence of AgNO_3_, the sonostimulation resulted in the typical DMPO-OH adduct, irrespective of the metal-oxide catalyst present in solution (Fig. 3A). This result confirms the production of the hydroxyl radical ·OH and the occurrence of water sonolysis (Eq. 2), as extensively reported in the literature(Serpone et al. 1994; Weavers et al. 1998; Mišík and Riesz 2000; Ince et al. 2001) (Fig. 3A):

**Fig. 4:**
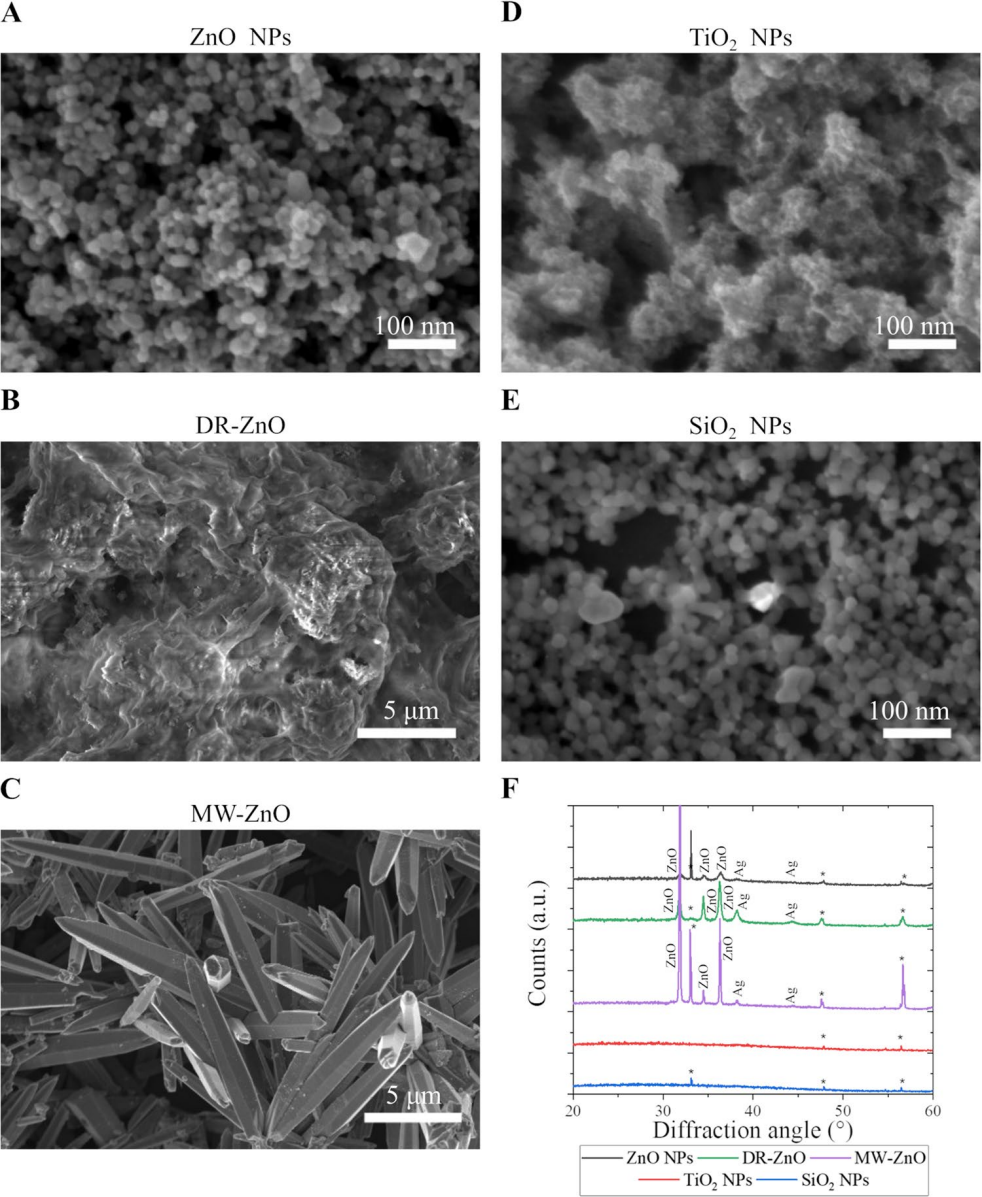
FESEM images of: (**A**) ZnO NPs, (**B**) DR-ZnO microparticles, (**C**) MW-ZnO microparticles, (**D**) TiO_2_ NPs and (**E**) SiO_2_ NPs after sonocatalytic stimulation in the AgNO_3_ water medium. (**F**) XRD patterns of the metal oxides after sonocatalytic irradiation in AgNO_3_ water solution. The asterisks * indicate the peaks related to the silicon substrate onto which the powders were deposited

2 $${H}_{2}O+US\to {H}^{.}+{OH}^{.}$$

However, when AgNO_3_ is added to the solution (Fig. 3B), the spectrum observed after the sonostimulation in presence of the ZnO particles is characteristic of the 5,5-dimethylpyrroline-(2)-oxyl-(1) (DMPOX), which is an oxidized form of DMPO, while it remains unvaried when the process occurs in presence of the other oxides, TiO_2_ and SiO_2_ and in AgNO_3_ solution without NPs. This is a clear indication that a strong oxidative environment is formed, very probably at the NPs surface, when the ZnO particles are coupled with AgNO_3_ salts and, most importantly, US radiation.

**Fig. 5:**
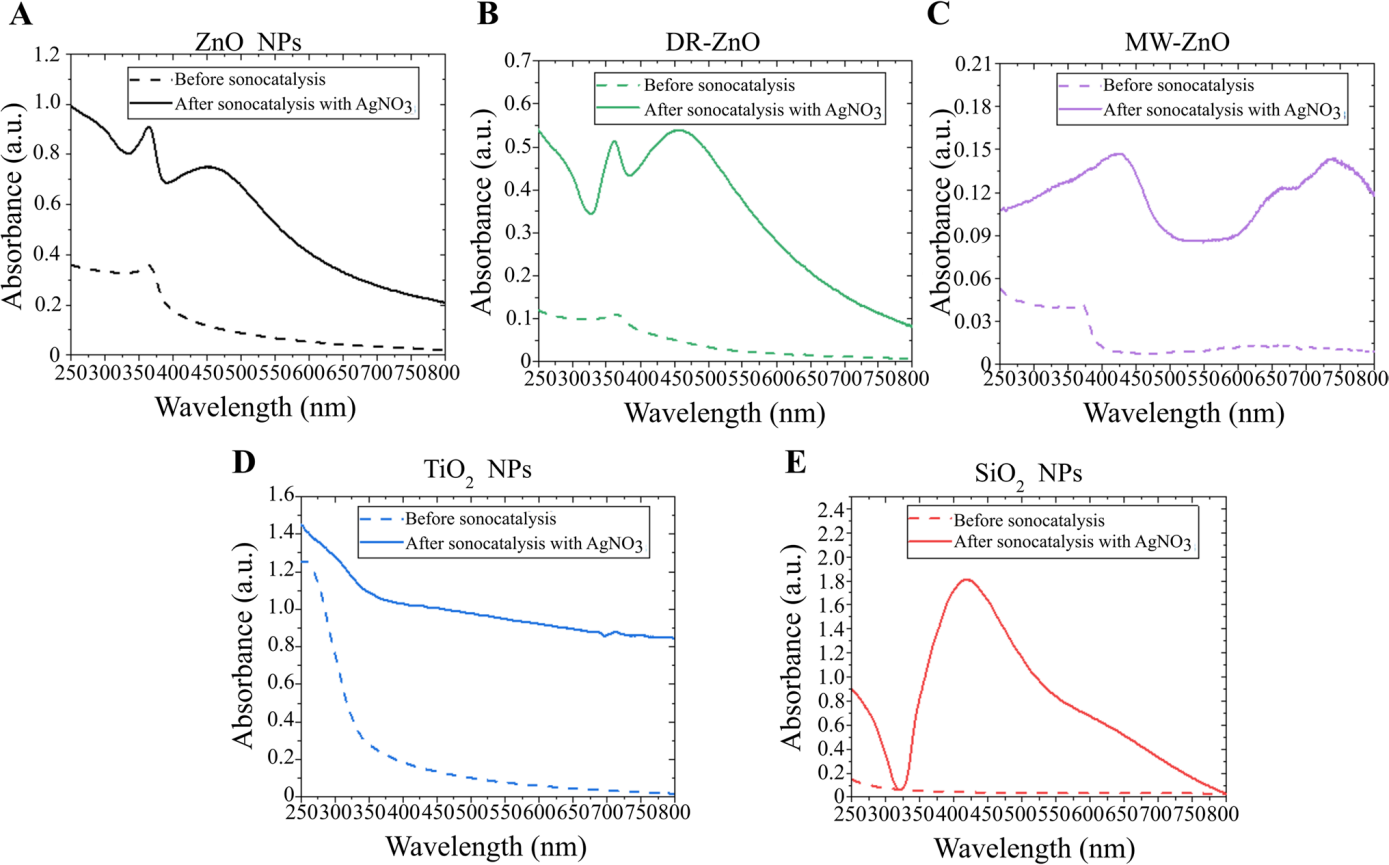
(**A**) ZnO NPs, (**B**) DR-ZnO microparticles, (**C**) MW-ZnO microparticles, (**D**) TiO_2_ NPs and (**E**) SiO_2_ NPs UV–vis spectra before and after sonocatalytic irradiation in AgNO_3_-containing water solution

Concerning the concentrations of the spin adducts, the amount of DMPO-OH (Fig. 3C) without AgNO_3_ in the solution does not change significantly among the different catalysts in these conditions, and none of them leads to an enhancement of the adduct concentration with respect to the one resulting from the sonostimulation of the aqueous solution only.

The addition of AgNO_3_ slightly reduces the amount of DMPO-OH formed in the aqueous solution in presence of SiO_2_ NPs, and more evidently when TiO_2_ NPs are used as catalysts (see dashed bars in Fig. 3C). As mentioned above, in the presence of ZnO the AgNO_3_ salt addition during the sonocatalytic stimulation results in the generation of the DMPOX adduct, which replaces completely the DMPO-OH production. The quantification of DMPOX production is depicted in Fig. 3D. It is clear that it is produced only from ZnO particles solutions and the DMPOX amount is lower for MW-ZnO microparticles than for DR-ZnO microparticles and ZnO NPs (Fig. 3D). The reason for this behavior may lay in the different surface area exposed by the different morphologies of the ZnO, with a larger amount of ROS generated with the system having the large surface area. This suggests that the majority of the phenomena occurs at the surface of the semiconductor.

As a further test to highlight the nature of the ROS generated during the sonostimulation of particles suspensions, the generation of singlet oxygen was measured with a specific fluorescent dye (SOSG). As it can be observed from Fig. 3E, the sonostimulation of aqueous suspension of all kinds of particles leads to fluorescence signals that are higher than the ones observed for pure water and AgNO_3_ solutions without any stimulation. This clearly means the generation of singlet oxygen from such water suspensions with a trend similar to what already observed for the EPR measurements. However, in presence of AgNO_3_ with particles, the signal is higher than the control one only in presence of ZnO, specifically ZnO NPs and ZnO DRs. This is in agreement with what is found with the EPR measurement, in which we have the production of DMPOX (Fig. 3D), probably due to a highly oxidative environment. In the cases where AgNO_3_ is not present, the generation of singlet oxygen is a relevant phenomenon, but the absence of an electron scavenger, probably leads to different phenomena that do not permit the oxidation of the DMPO trap in the EPR measurements.

### Characterization of the oxides after sonostimulation in presence of AgNO_3_

After ROS generation tests, the sonostimulated particles in presence of AgNO_3_ were deeply characterized to understand the mechanism behind the different spin-adduct obtained with the ZnO nano and microparticles only.

Firstly, the stimulated particles were investigated in terms of morphology and elemental composition by means of FESEM and EDS, respectively. Figure 4 reports the representative FESEM images of the metal oxides particles analyzed after their sonostimulation in the presence of AgNO_3_. The particles morphology of ZnO NPs and MWs is not modified in a major way, while DR-ZnO microparticles show a coating layer deposited on their surface. It cannot be excluded the presence of a coating onto the particles derived from the reaction of silver ions and electrons (e.g. Ag^+1^ that is reduced to Ag^0^) at the particles surface. Indeed, a very conformal surface coating would not influence the overall morphology of the particle in all the examined cases and can be probed only by elemental analysis EDS (Table 2).

**Table 2:** EDS analysis of the metal oxides after sonostimulation in the AgNO_3_ water solution (atomic percentages)

	Zn (At. %)	Ti (At. %)	Si (At. %)	O (At. %)	Ag (At. %)
ZnO NPs	46.12 ± 1.16	-	-	51.91 ± 0.54	1.97 ± 0.62
DR-ZnO	33.55 ± 1.02	-	-	63.16 ± 5.39	3.28 ± 0.06
MW-ZnO	48.20 ± 0.74	-	-	49.57 ± 0.55	2.22 ± 0.18
TiO2 NPs	-	17.92 ± 1.82	-	75.67 ± 2.18	6.41 ± 0.37
SiO2 NPs	-	-	21.95 ± 0.61	75.01 ± 1.22	3.03 ± 0.61

In all the analyzed particles, it is indeed possible to detect Ag, coming from the AgNO_3_ dissolved in the water suspension of the particles. For ZnO NPs and MW-ZnO particles, the low amount of silver present in the systems does not significantly affect the stochiometric ratio between zinc and oxygen. Furthermore, this low level of Ag does not allow to determine whether the AgNO_3_ has crystallized on top of the particles or whether silver oxide or metallic silver was formed. For silica, titania and DR-ZnO microparticles, the ratio between the Zn and O elements composing the metal oxide structure excesses the stoichiometry, in favor of a higher amount of oxygen in all cases. This higher value of oxygen could come either from the silicon substrate (where the particles are deposited to perform the EDS measurement) or from possible AgNO_3_ residuals.

To assess the nature of silver compounds formed during sonostimulation and possible difference among the various particles, the XRD patterns of the oxides stimulated in AgNO_3_ solution were considered (Fig. 4F). As it can be seen, no major modifications on the crystalline structure of the various oxides are induced during this process. Interestingly, in all the XRD patterns of ZnO particles it is possible to spot the presence of two peaks of metallic silver at the diffraction angles of approximately 38° and 44°, corresponding to the (111) and (200) crystalline planes of silver, respectively, according to ICSD n. 044387 (Card n. 893,722). In contrast, the XRD patterns of titania and silica NPs do not report any further peak which can be attributed to a crystalline phase of silver.

However, the presence of the Ag element in the samples composed by SiO_2_ and TiO_2_ suggests either that a small amount of AgNO_3_ (bellow the detection limit of the XRD analysis) remained adsorbed on the particles surface or that an amorphous silver compound has formed also in these samples.

The UV–vis absorption spectra of the US exposed metal-oxides particles in presence of AgNO_3_ are reported in Fig. 5. Interestingly, in all the cases it is possible to spot the rise of new absorption peaks at higher wavelengths than the typical ones of the oxides. The position of the peak changes upon the considered particles and there is also a difference between the various morphology of ZnO particles. In general, for ZnO NPs, SiO_2_ NPs and DR-ZnO microparticles the peak is centered close to 450 nm with a broadening that takes approximately 100 nm. The MW-ZnO spectrum exhibit the rise of a new peak centered close to 700 nm. The reason for this behavior may be attributed to the formation of a layer of silver-related chemical compounds. More in detail, the presence of metallic silver, that is also suggested by the XRD patterns, could provide some plasmonic resonances at the particles surface that may be responsible for the new absorption peaks. As already reported, the position of these plasmonic peaks depends from the dimension of the silver clusters themselves(Liu et al. 1998). Finally, the TiO_2_ NPs present a general increase in the absorbance, but there are no new peaks that clearly appear. This fact suggests that silver is not formed in this system. For silica NPs, it is possible to find a new, well resolved peak that suggests the formation of amorphous silver clusters or coating on these SiO_2_ NPs, similar to what found in several previous works(Ung et al. 1999; Kobayashi et al. 2005; Choi et al. 2013).

Apart from the elucidation of the mechanism involved during the sonostimulation, the result here reported represents a promising way to deposit silver on the surface of the NPs with a substantially conformal surface coating that can be exploited when the synthesis of a core–shell nanoparticle is required.

### Sonocatalytic water oxidation evaluation through gas-chromatography

To determine the potentialities of the particles considered as oxygen producer in hypoxic conditions, we measured the oxygen concentration in a small reactor chamber during an oxygen-depleted water solution sonostimulation. A water solution containing the particles was placed in the chamber and stripped with argon. Then, the oxygen concentration of the gas above the solution was measured during US stimulation for 1 h. In this way it is possible to evaluate the influence of the metal oxide in the oxygen generation in water. The measurements were repeated in presence of AgNO_3_ salt.

**Fig. 6:**
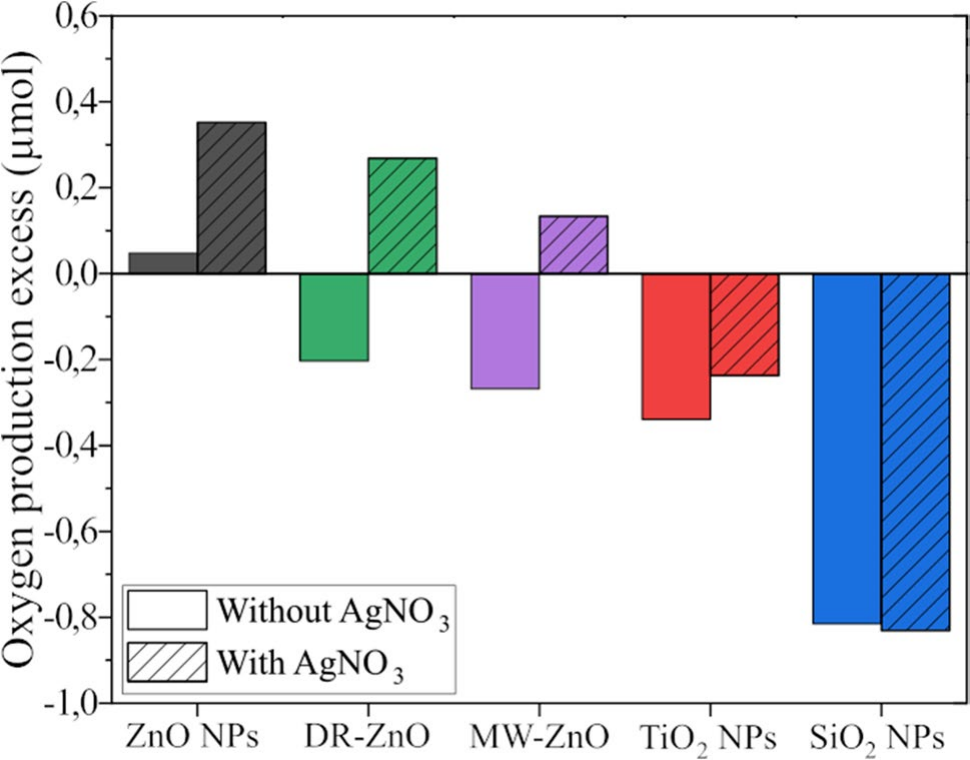
Measured oxygen production excess (with respect to reference measurements performed in simple water), when the metal oxides particles suspensions are US stimulated in presence or in absence of AgNO_3_

The evaluation of the amount of oxygen produced during the US stimulation of the different oxides has been carried out in the reactor described in Section 2.3. Figure 6 reports the relative increments of oxygen concentration excess produced with respect to the amount obtained by sonostimulating water without any particle and AgNO_3_. For ZnO NPs, the oxygen production without electron scavenger is very close to the one obtained in pure water. An increase of the oxygen production is observed in the presence of AgNO_3_. ZnO microparticles, i.e. DR-ZnO and MW-ZnO, present similar results. Indeed, when they are stimulated with ultrasound in simple water without the AgNO_3_ salt, the oxygen production is reduced with respect to the control sample composed of simple water, presenting a negative oxygen concentration excess. However, when AgNO_3_ is added to the system, there is an increase of oxygen production, which suggests that ZnO microparticles coupled with ultrasound and electron scavenger are able to mediate a process that leads to molecular oxygen production. Conversely, the oxygen excess is negative for both TiO_2_ and SiO_2_ NPs, with and without electron scavenger, indicating that oxygen production is inhibited in both cases by the presence of these NPs.

Apparently, only ZnO micro and NPs coupled with AgNO_3_ were able to increase the occurrence of water oxidation during sonostimulation. This trend resembles somehow the one found for ROS production. Indeed, ZnO NPs present the largest oxygen excess and are coupled with a large amount of generated DMPOX. This amount is similar to the one obtained from DR-ZnO microparticles which can be ranked in second place in terms of oxygen excess production. Finally, MW-ZnO particles still present a positive oxygen excess production but lower than the other two systems, probably because of the lower surface area exposed. The associated DMPOX concentration is also low. Apart from the internal differences found between the ZnO particles, in general the results obtained from the different oxides, in presence or absence of AgNO_3_, indicate that different phenomena occur, especially when ZnO is used together with AgNO_3_.

### Live/Dead assay

As a proof of concept, ZnO particles were tested on a biological system that could benefit from ROS and oxygen generation via remote stimulation. The test focused on ZnO particles only, as they showed the best ROS generation performance under various conditions. AgNO_3_ was excluded due to its nonspecific cytotoxicity(Hidalgo and Domínguez 1998), but the presence of several ions in biological systems can also act as scavengers similar to silver ones. In this way, our experiment can offer a further proof, being aligned to a real case scenario.

**Fig. 7:**
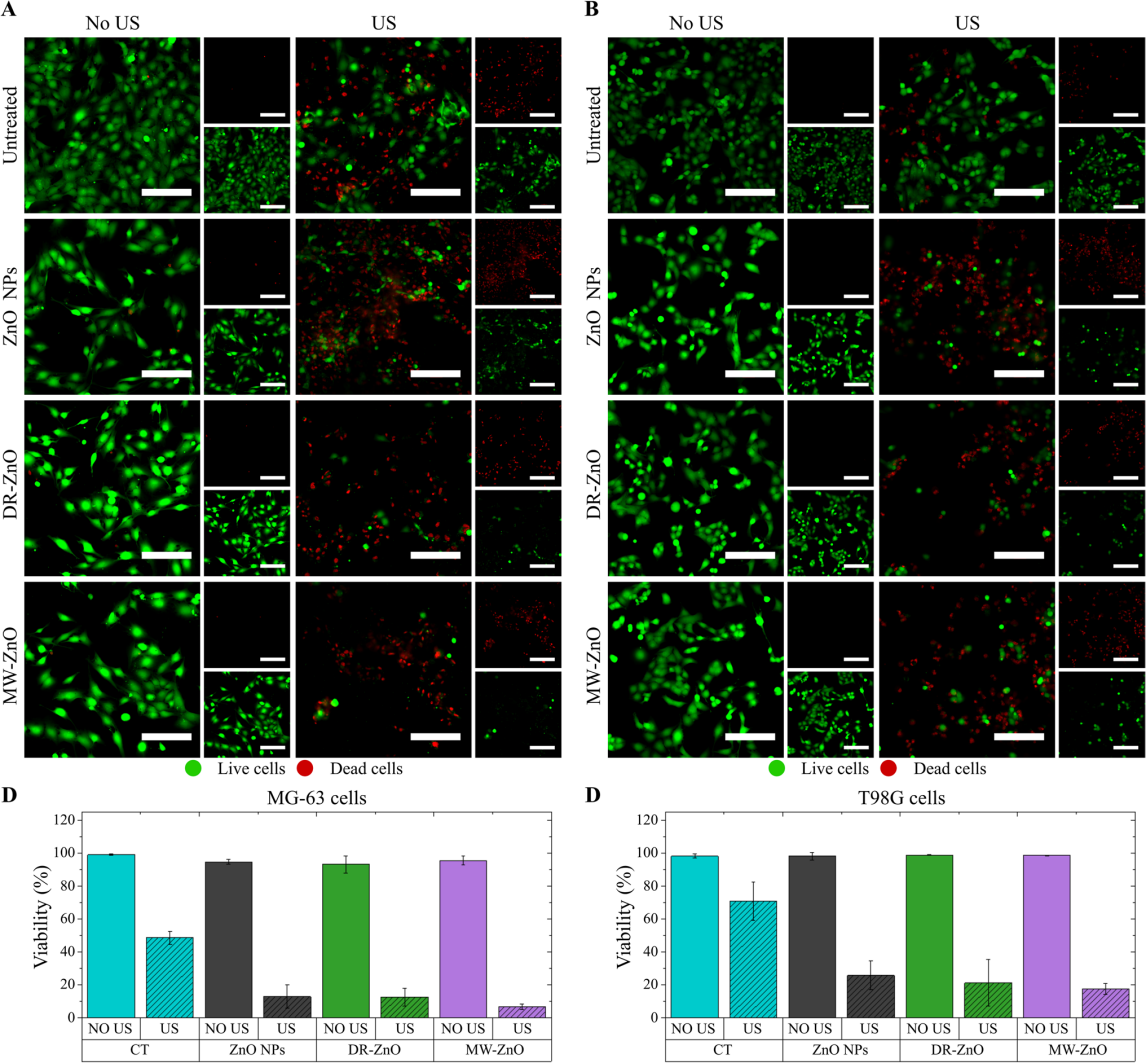
(**A**) Representative fluorescence microscopy images of the Live/Dead assay performed after the sonostimulation for osteosarcoma MG-63 (**A**) and glioblastoma T98G (**B**) cells. Scalebar: 200 µm. The green signal is related to the live cells, the red signal is related to the dead cells. Quantification of the cells viability based on the results of the Live/Dead assay for MG-63 (**C**) and T98G (**D**) cells

Figure 7A shows that untreated cells (no ZnO nor US) exhibited no toxicity, as indicated by the high abundance of green signal. Similarly, cells belonging to both the analyzed cell lines (osteosarcoma MG-63 and glioblastoma T98G) and treated with 8 µg/mL ZnO showed no toxicity, despite the known dose-dependent toxicity of nanosized ZnO(Carofiglio et al. 2020), which is avoidable at low concentrations as the one used in this case.

US stimulation alone was slightly toxic for both the cell lines, as evidenced by red dots representing dead cells in Fig. 7A and 7B. A higher viability for the US treated cells is found for T98G cells, indicating a possible higher resistance of this cell line toward US stimulation. However, live cells remained in both cell lines, suggesting that US is insufficient to completely suppress tumor growth. When ZnO particles were combined with US treatment, toxicity peaked, reducing live cell numbers significantly and altering their morphology to a rounded shape, indicating detachment from the surface. Quantification (Fig. 7C and 7D) confirmed this, showing that the viable cell percentage after combined ZnO-US treatment was close to 10% for osteosarcoma cells and 20% for glioblastoma cells.

## Summary and proposed mechanism

To determine which are the causes of the reported behaviors, we summarize here the findings and propose a hypothesis for the mechanism at the base of the phenomenon observed in this work. Indeed, from the results reported above, the following experimental evidence can be listed:

- molecular oxygen is produced in excess with respect to ultrasonically-treated pure water system and freed in gaseous form upon the ultrasonic stimulation of water solutions containing both ZnO and AgNO_3_. In all the other cases explored in this work, no excess O_2_ production is in contrast measured (see Fig. 6).
- After ultrasound irradiation in the presence of AgNO_3_, all metal oxides nano- and microparticles reveal the presence of elemental silver from the EDS analyses (see Fig. 4 and Table 2), accounting to the precipitation of a silver chemical compound. In all the tested ZnO particles, XRD patterns confirmed the formation of crystalline metallic silver, while SiO_2_ and TiO_2_ NPs do not present the same peaks (Fig. 4F), suggesting the precipitation of amorphous species. The presence of a novel absorption peak in the UV–vis absorption spectrum of the SiO_2_ NPs, similar to the one found on the ZnO particles, indicates the precipitation of reduced silver species also with SiO_2_ NPs (Fig. 5).
- ROS generation was reported for all the tested metal oxide micro and nanostructures during ultrasound irradiation in water media (see Fig. 3). In particular, in the absence of AgNO_3_, the DMPO spin trap reveals the presence of DMPO-OH adducts deriving from important levels of water sonolysis and thus the generation of OH· radicals according to Eq. 2(WANG and XU 2012).
- It is however of note that the presence of AgNO_3_ in water solution modifies the generated ROS forming a specific oxide species of the employed spin-trap. This happens only when the sonocatalyst is ZnO. In this case, the DMPO-OH adduct is not obtained (as for pristine water with AgNO_3_, TiO_2_ or SiO_2_ NPs), but the new spin adduct DMPOX is detected by EPR spectroscopy. DMPOX is the oxidized version of DMPO, indicating that, only in presence of ZnO particles together with the AgNO_3_ salt, a considerable amount of oxidizing species different from the ones formed in presence of TiO_2_ and SiO_2_ are produced as a result of sonostimulation.

Given the above results, it is possible to formulate different hypotheses. A first hypothesis for the generation of oxygen under the sonostimulation of metal-oxide containing solution could be related to the semiconductive behavior of the considered metal oxides. Indeed, under ultrasound irradiation, the microbubbles could collapse and generate, as already mentioned, sonoluminescence(Crum 2015). This sonoluminescence, whose bandwidth takes all the visible spectrum of light and also part of the UV region(Vighetto et al. 2022), could in principle excite the electrons of the semiconductors (i.e. both ZnO and TiO_2_ particles) from the valence band to the conduction band. This phenomenon could in principle be a very powerful tool in cancer therapy, since it would allow to have a localized photodynamic therapy that overcome the limits of poor tissue penetration of the light used in conventional photodynamic. Moreover, the photogenerated electrons can reduce Ag^+^ into metallic silver, leaving sufficient holes for water oxidation and, consequently, oxygen production, only when AgNO_3_ is dissolved in the solution. If this finding would explain the precipitation of metallic silver on the semiconductor particles surface, it does not explain why TiO_2_ NPs do not show any oxygen excess production. The phenomenon could be in principle related to the photocorrosive behavior of ZnO, that could allow for the production of oxygen as a result of the corrosion of ZnO by the photogenerated holes(Zhang et al. 2009; Dworschak et al. 2020). However, the phenomenon should not depend on the presence of silver ions, as observed experimentally. Moreover, the EDS and UV–vis analyses of SiO_2_ exhibit the precipitation of silver species on its surface, showing again that, despite being a process that is very likely to occur, it is surely not the only one. An explanation for the presence of silver also on inert SiO_2_ NPs can come from the study of Pol et al.(Pol et al. 2002). The authors managed to cover silica spheres with silver NPs by means of AgNO_3_ coupled with US stimulation and ammonia (the latter exploited to activate the silica surface to allow a proper coating) in an inert atmosphere. In their work, they proposed water pyrolysis due to US stimulation (Eq. 2) as the main cause for the reduction of Ag^+^ ions that, according to their work, is mediated by the hydrogen radical (Eq. 3):

3 $${Ag}^{+}+ {H}^{\cdot }\to {Ag}^{0}+{H}^{+}$$

The result was an amorphous silver coating that required further annealing for the crystallization. This is very similar to what found for our SiO_2_ NPs, in which we observe the reduction of Ag^+^. It can be easily extended to both ZnO and TiO_2_ particles results, being the general phenomenon not specifically dependent on the nature of the considered particle. Furthermore, the presence of AgNO_3_ residuals cannot be completely excluded.

The mechanisms described in Eq. 2 and 3 do not however explain the difference in the oxygen production during sonostimulation among the various oxides and the silver crystallinity found only on ZnO particles for which an excess of oxygen production is measured.

The only aspect that has not been considered so far is the piezoelectricity of ZnO. Indeed, ZnO is the only piezoelectric material considered in this work, meaning that it has the ability to convert a mechanical stimulus into an electrical potential. It has been already shown that ZnO can induce water splitting upon US stimulation(Hong et al. 2010). The hypothesis supported by the literature is that ZnO microfibers are polarized due to the mechanical stress induced by the ultrasound radiation. The surface polarization is then able to generate a sufficient potential to split water molecules, without any salt addition. However, we observed that the addition of AgNO_3_ is necessary for water oxidation. Therefore, according to our experimental results, we hypothesize that, once polarized by the US stimulation, the ZnO particle negative face act as anode and the positive one as cathode of a nano-electrolytic system immersed into an electrolytic solution of AgNO_3_. More in detail, when the ZnO particles is subjected to the US radiation, it polarizes to provide electrons to the Ag^+^ ion, reducing it to metallic silver. This prevents the holes from recombination, so that they can oxidize water and generate molecular oxygen according to Eq. 1 (Santos et al. 2013; Thalluri et al. 2013; Sen et al. 2022). Moreover, Ag^+^ is known to have a standard reduction potential of 0.7993 V, higher than the one of H^+^ (0 V)(Bratsch 1989). Therefore, when the salt is not present in the solution, a higher potential is required to trigger the reaction and capture electrons in the process. Probably, the piezopotential generated by means of ultrasound stimulation is not sufficient for the overall water splitting reaction to occur in absence of Ag^+^ ions, explaining why oxygen production is not observed during ZnO sonostimulation in pure water. Moreover, since the H^+^ produced during water splitting diffuse very slowly in pure water, they are not able to reach the electrons in the other pole of the ZnO material to be transformed into H_2_. The electrons and holes are more likely to recombine in absence of Ag^+^ ions. The main phenomenon occurring remains inertial cavitation, as it happens for SiO_2_ and TiO_2_ NPs. Inertial cavitation results in the generation of hydroxyl radicals that seem to not sensibly contribute to the excess of oxygen production, but still provide the conditions for the formation of amorphous silver as previously described.

This hypothesis is further corroborated by the results obtained from the EPR analysis, in which the oxidative power of the US treatment is only expressed in presence of both AgNO_3_ and ZnO (DMPO is oxidized in DMPOX) while, with SiO_2_ and TiO_2_ NPs, as well as with ZnO in absence of the electrolyte (AgNO_3_), hydroxyl radicals are always produced.

As a final remark, the morphology and aspect ratio of the ZnO particles does not seem to have a substantial effect on the occurring phenomena, with MW-ZnO microparticles presenting a slightly lower oxygen production than the other two morphologies, probably due to a smaller active surface area.

Interestingly, the combined ZnO-US treatment showed high efficacy against an osteosarcoma and glioblastoma cell line in vitro, indicating the high significance of choosing ZnO as both semiconductor and piezoelectric material. It is worth noting that the cell culture medium contains salts and biological moieties that may act as electron scavengers, similar to silver ions. This allows ZnO to maintain a high oxidative potential and facilitates localized oxygen production and ROS via remote stimulation, imparting cell viability.

## Conclusion

In the present work we report on the potential use of metal oxide micro- and nanoparticles as catalysts to assist water oxidation when coupled with an ultrasound stimulation for cancer treatment. Three metal oxides were considered: (i) optically and electromechanically inert SiO_2_ NPs; (ii) electromechanically inert TiO_2_ NPs and (iii) piezoelectric ZnO NPs. To also take into account the influence of the particle morphology in the occurrence of water oxidation, ZnO in the form of nanoparticles and microparticles, having either a microwire or flower-like morphology, were used and tested. After an optical and morphological characterization, the particles were immersed in an aqueous solution and stimulated with ultrasound. The process was analyzed in terms of radical formation during sonostimulation with EPR measurements. For ZnO in pure water, and for SiO_2_ NPs and TiO_2_ NPs in both pure water and in presence of Ag^+^ the formation of hydroxyl radicals is detected, as widely reported in the literature. In contrast, for ZnO systems with Ag^+^ ions, the oxidized version of the spin trap was detected. Electron microscopy images, elemental analyses, and X-ray diffraction patterns allowed to detect the presence of crystalline silver only on the ZnO surface after sonostimulation with AgNO_3_, while small residues of amorphous silver and AgNO_3_ were found on both SiO_2_ and TiO_2_ NPs, suggesting a different precipitation mechanism in these cases, with interesting potentialities in the field of surface coating. Finally, the amount of molecular oxygen was measured during the sonostimulation. The experiment was repeated adding AgNO_3_ to the solution to provide Ag^+^ ions. From the results, it is possible to observe a value of the produced oxygen higher than the pure water one only for the systems in which both Ag^+^ ions and ZnO particles, regardless of morphology, were present, in parallel to the formation of DMPOX and singlet oxygen in the ROS generation measurements. The mechanism that we propose is based on the piezoelectricity of ZnO, which allows the particles to polarize under mechanical stimulation. The Ag^+^ can reduce and act as electron scavenger to prevent holes recombination which can, in turn, act as oxidizing agents for water. If AgNO_3_ is not included in the reaction, the potential generated at the faces of the piezoelectric particle is not sufficient for the reaction to occur. This can be reconducted to the fact that the electric potential required to reduce H^+^ to H_2_ is higher than that of Ag^+^ to Ag. Also, the coexistence of inertial cavitation cannot be excluded, which, according to the literature, is responsible for the generation of hydroxyl radicals and the precipitation of amorphous silver on the particles, without participating in the production of oxygen.

Finally, the ZnO particles were tested against an osteosarcoma and glioblastoma cell lines, which can be efficiently killed by the ZnO-US combined treatment. This proof-of-concept results confirms the great premises for the exploitation of metal-oxide piezoelectric nanosystem for sonodynamic therapy.

## Supplementary Information

Below is the link to the electronic supplementary material.

Supplementary file1 (DOCX 543 KB)

## Data Availability

Data is provided within the manuscript or supplementary information files.
