# Supplementary material for: Ultrasound-assisted water oxidation: unveiling the role of piezoelectric metal-oxide sonocatalysts for cancer treatment
Source: Biomed Microdevices. 2024 Aug 19;26(3):37. doi: 10.1007/s10544-024-00720-3 (PMC11333555; doi:10.1007/s10544-024-00720-3)
Supplement: Supplementary file 1 — Supplementary file1 (DOCX 543 KB) [file 10544_2024_720_MOESM1_ESM.docx]

Supporting information

Ultrasound-assisted water oxidation: the role of piezoelectric metal-oxide sonocatalysts

Marco Carofiglio^1^, Nicolò Maria Percivalle^1^, Simelys Hernandez^1^, Marco Laurenti^1^, Giancarlo Canavese^1^, Joana C. Matos^2^, M. Clara Gonçalves^3,4^, Valentina Cauda*

1. Department of Applied Science and Technology, Politecnico di Torino, C.so Duca degli Abruzzi 24, 10129 Turin, Italy

2. CESAM - Universidade de Aveiro, Campus Universitário de Santiago, Aveiro, Portugal

3. CQE, Centro de Química Estrutural, Universidade de Lisboa, Av. Rovisco Pais, IST, 1000 Lisboa, Portugal

4. Departamento de Engenharia Química, Instituto Superior Técnico, Universidade de Lisboa, Av. Rovisco Pais, 1000 Lisboa, Portugal


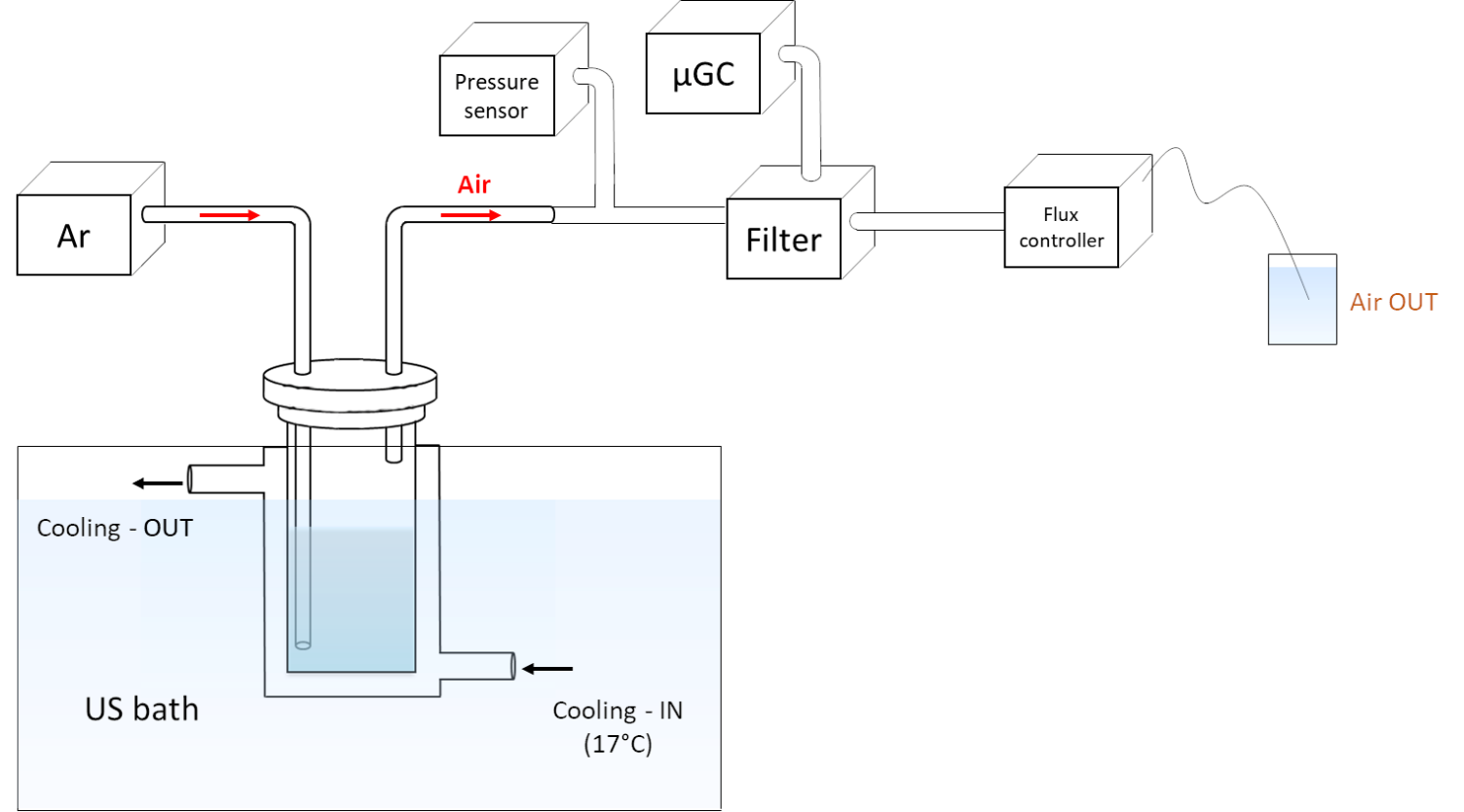


**Figure S1.** Experimental setup for oxygen production measurement.


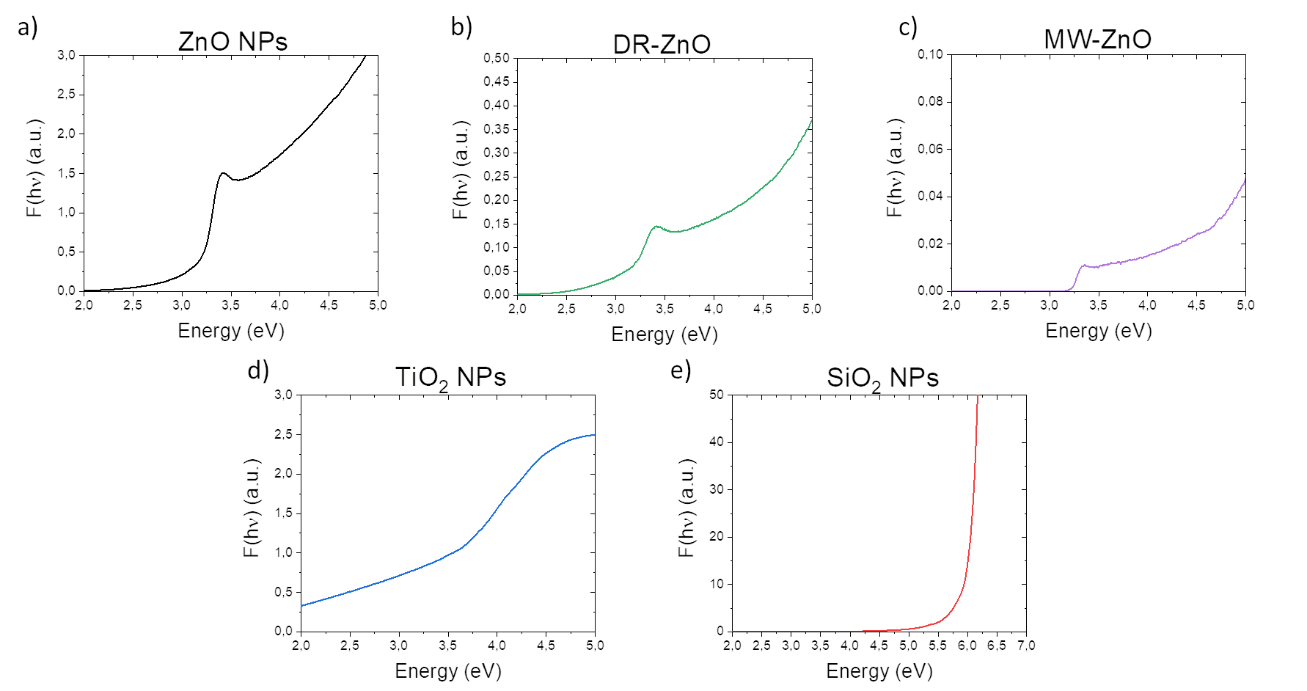


**Figure S2.** Tauc’s plots, respectively, for: (a) ZnO NPs, (b) DR-ZnO microparticles, (c) MW-ZnO microparticles, (d) TiO_2_ NPs and (e) SiO_2_ NPs.


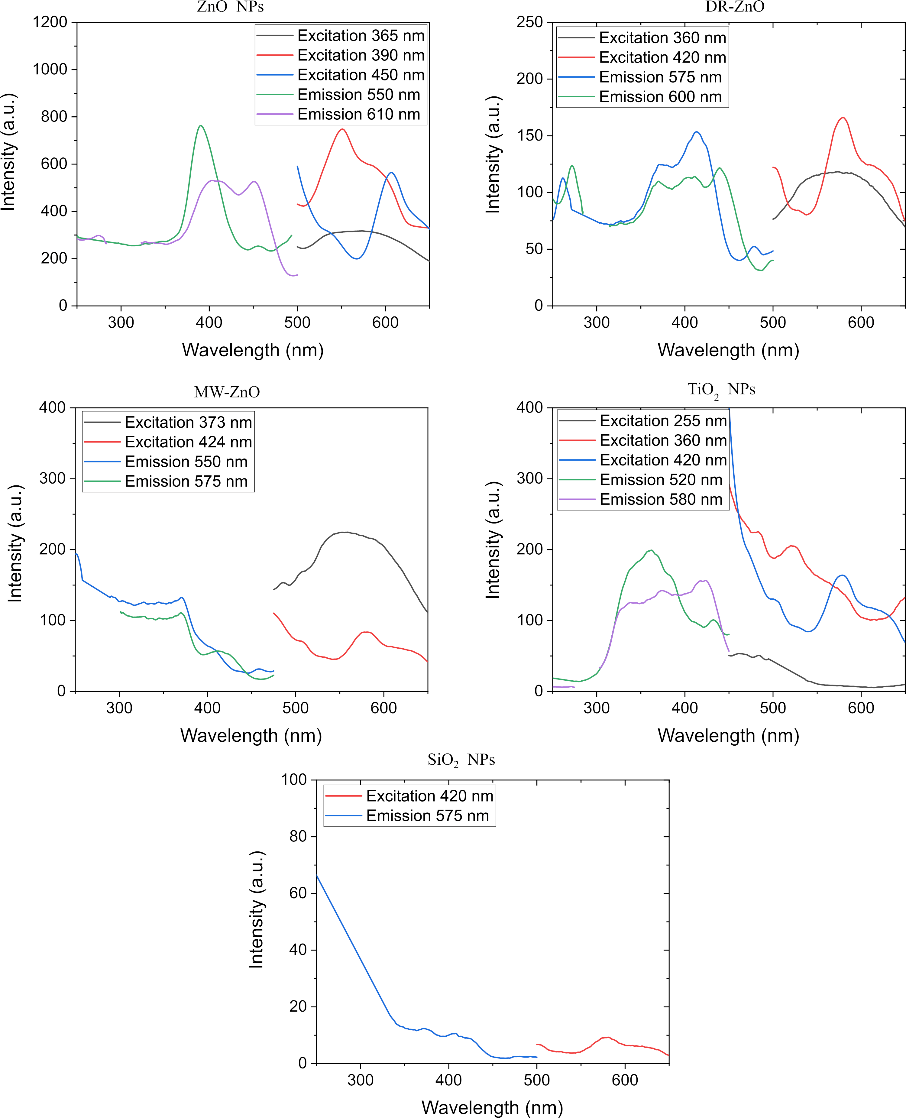


**Figure S3.** Fluorescence excitation and emission spectra acquired at different emission and excitation wavelengths, for: ZnO NPs, DR-ZnO microparticles, MW-ZnO microparticles, TiO_2_ NPs and SiO_2_ NPs.
